# Alternative Pathway Involvement in Protoporphyria Patients Related to Sun Exposure

**DOI:** 10.3389/fimmu.2021.615620

**Published:** 2021-02-16

**Authors:** Francesca Granata, Lorena Duca, Valentina Brancaleoni, Silvia Fustinoni, Giacomo De Luca, Irene Motta, Giovanna Graziadei, Elena Di Pierro

**Affiliations:** ^1^Fondazione IRCCS Ca' Granda Ospedale Maggiore Policlinico, U.O.C. Medicina Generale, Milan, Italy; ^2^EPIGET - Epidemiology, Epigenetics, and Toxicology Lab, Department of Clinical Sciences and Community Health, Università degli Studi di Milano, Milan, Italy; ^3^Environmental and Industrial Toxicology Unit, Fondazione IRCCS Ca' Granda Ospedale Maggiore Policlinico, Milan, Italy; ^4^Department of Clinical Sciences and Community Health, Università degli Studi di Milano, Milan, Italy

**Keywords:** protoporphyria, complement system, alternative pathway, phototoxic reaction, global radiation, UV detection

## Abstract

The homeostasis of tissues in a chronic disease is an essential function of the alternative pathway (AP) of the complement system (CS). However, if not controlled, it may also be detrimental to healthy cells with a consequent aggravation of symptoms. The protoporphyria (PP) is a rare chronic disease that causes phototoxicity in visible light with local skin pain and general malaise. In order to establish if there is a systemic involvement of the CS during sun exposure, we designed a non-invasive method with a serum collection in winter and summer from 19 PP and 13 controls to detect the levels of CS protein: Properdin, Factor H (FH), and C5. Moreover, the global radiation data were collected from the regional agency of environmental protection (ARPA). The results show growing values for every protein in patients with PP, compared to control, in both seasons, in particular in summer compared to winter. To reinforce the evidence, we have estimated the personal exposure of patients based on the global radiation data. The main factors of the AP increased over the season, confirming the involvement of the AP in relation to light exposure. The systemic response could justify the general malaise of patients after long light exposure and can be exploited to elucidate new therapeutic approaches.

## Introduction

The complement system (CS) comprises a network of 50 proteins, and being a part of the innate immune system, it can be activated through three different pathways: classical, lectin, and alternative (also known as properdin pathway) (1).

Several investigations over the last decade tried to decipher the function of the alternative pathway (AP) in inflammation (2), especially in a chronic disease (arthritis, asthma, and kidney failure). Active participation of this pathway has been well-established in regulating the homeostasis of tissues, eradicating cellular debris, orchestrating immune responses, and sending “danger” signals (3–5). However, if the AP of the CS becomes uncontrolled, it can also attack the healthy cells, resulting in tissue destruction and aggravation of symptoms in the acute phase of some diseases [stroke, atypical hemolytic uremic syndrome (aHUS), or primary thrombotic microangiopathy (TMA)] (6–9). Properdin, a multimeric protein, takes part in the activation of the AP by acting as a positive regulator of the amplification loop (10). Properdin possesses a strong binding affinity for C3b, originating from the spontaneous hydrolysis of C3 protein in the fluid phase (**Figure 5**) (11, 12). Under physiological conditions, the hydrolyzed form of C3 has a short half-life (~90 s), both in the fluid phase and on cell surfaces (13). The C3 and C5 convertase complexes are stabilized upon binding with properdin and the Factor B (FB; other protein of the AP) that increase their half-life by 5–10-fold (2). This mechanism leads to the activation of the AP loop solution. Another important phenomenon involved in the regulation of AP is the Factor H (FH) competing with FB for binding to C3b, in turn, inhibiting the assembly of the C3 convertase and facilitating the degradation of the already formed complex (14). The protective mechanism thus functions as a sentry to ensure that only when it is required, the spontaneous activation of C3 is stabilized by properdin and FB with a consequent stabilization of the C3b subunit and activation of C5 (3).

Erythropoietic protoporphyria (EPP; OMIM 177000) and X-linked protoporphyria (XLP; MIM 300752) are two rare disorders, triggered, respectively, by the deficiency of the enzyme ferrochelatase (FECH; EC 4.99.1.1) or the activation of the erythroid-specific form of 5-aminolevulinate synthase 2 (ALAS2; EC 2.3.1.37) in the heme biosynthesis pathway (15, 16). The exacerbation of the symptoms is due to the accumulation of protoporphyrin-IX (PP-IX) in erythrocytes and tissues, in particular in dermis, and consists of a severe photo reaction. The PP-IX is excited at 410 nm in the Soret band of visible light, with significant emission peaks of activation at 635 nm (**Figure 3A**) (17). The visible spectrum partially overlaps the UVA (from ~360–380 to 400 nm in the deep violet) ending at 780 nm (**Figure 3A**) (18). Thus, the PP-IX peak shows its ascending curve in this region between late UVA and the visible light spectrum, with its maximum peak at 410 nm. The visible light of global solar radiation is not limited to the wavelengths in the visible limit, but it includes the total short-wave radiation falling from the sky onto a horizontal surface on the ground. Also, it includes both the direct solar radiation and the diffuse radiation resulting from reflected or scattered sunlight (19).

Within few minutes of exposure to this wavelength, individuals with this disease develop extremely painful cutaneous conditions and the symptoms include burning, acute skin pain, itching, and edema (20).

The biological process that leads to a phototoxic reaction in patients with PP has not yet been completely elucidated. It is widely known that the phototoxic reaction occurs because of the increased level of reactive oxygen species (ROS) in the derma, which leads to endothelial cell photodamage (21) through CS activation and mast cell degranulation, culminating into exocytosis of vasoactive mediators and acute inflammation (22).

The detection of the CS proteins, C3 and C5, in blood samples of only two patients with PP, collected after skin irradiation (0.7 J/cm^2^ at 400–410 nm), confirmed the involvement of the CS in the pathophysiology of PP symptoms (23). Our recent study, conducted through the assessment of two specific proteins, FB for the AP and C1q from the classical pathway, excluded the involvement of the latter, through the detection of the normal level in both seasons of C1q protein. Enhanced levels of FB in summer suggested the involvement of the AP in the molecular mechanisms, leading to a phototoxic reaction (24).

Considering the previously obtained data on FB and C3, the present study aimed to estimate in detail the involvement of the AP through the assessment of the properdin, FH, and C5 for in-depth knowledge of phototoxic reaction in patients with PP. Furthermore, through the collection of global radiation data, we estimated personal exposure to global radiation in patients with PP in order to better investigate the relationship between exposure and the metabolites of the AP.

## Materials and Methods

### Patients

The study involved a total of 19 patients with PP diagnosed and referred to the Rare Diseases Center at Foundation IRCCS Ca'Granda Policlinico of Milan and 13 healthy individuals aged-matched and randomly collected during the seasons. To verify the reaction to light exposure in each group, with any further invasive treatment, blood samples were collected during a routine test at two points in winter (January, February, and first week of March 2017) and summer (June, July, or the first week of August 2017), respectively. Routine blood panel analyses included specific porphyria parameters as erythrocyte-free PP-IX; zinc protoporphyrin (ZPP); and plasma peak performed following the methods previously described (25). According to the World Medical Association's Declaration of Helsinki for medical research, all subjects involved in this study signed informed consent for the diagnosis and research approved by the ethics committee of our institution, Fondazione IRCCS Ca' Granda Ospedale Maggiore Policlinico, and the identity of the study participants were anonymized.

### Laboratory Testing for C5, Properdin, and Factor H

Serum was separated from whole blood by centrifuging at 3,000 rpm at 4°C for 10 min and stored at −80°C. Serum C5, Properdin, and FH were determined using a commercial immunoassay kit (Hycult Biotech) following the manufacturer's indications (Hycult Biotech, Uden, the Netherlands). The two different samples from patients with PP were used to examine each marker, and the results were compared to values registered in healthy subjects.

### Global Radiation (GR) Data and UV Data

The service “IdroNivoMeteo e Clima” of Agenzia Regionale per la Protezione Ambientale–ARPA Lombardia provided the solar global radiation data from January 2017 to August 2017 for the Lombardy area. The data were transmitted as daily average radiation (MJ/m^2^). Moreover, the raw data of UVA (W/m^2^), from the different weather stations in Lombardy, expressed in W/m^2^ were given. The mean radiation received by each patient in the 14 days preceding the blood draw was calculated and used as a surrogate of personal exposure to global radiation (MJ/m^2^).

### Statistical Analysis

The data analysis was executed using GraphPad Prism software version 7 (2018 GraphPad 7.0e Software, Inc, USA). The D'Agostino–Pearson's normality test, the Shapiro–Wilk normality test, and the KS normality test were applied to confirm the normality of data from each measurement. In order to identify the outliers, the ROUT method (1%) was performed for each experiment. Unpaired or paired parametric *t*-tests and the Mann–Whitney *U*-test were employed for statistical analyses between seasons (winter vs. summer) and controls. Pearson's correlations were determined between two datasets with a two-tailed test and confidence interval at 95%. Linear regression was also calculated at a confidence interval of 95%. Box plots were used to represent the distribution of biological parameters at different quartiles of personal exposure to global radiation; ANOVA was used to compare the groups.

## Results

### Characteristics of Patients

The clinical and biochemical findings for patients involved in the study are summarized in Table 1. Nine EPP males, aged 39 ± 10.4 years, and 10 females, 39 ± 10.3 years, with PP were involved in this study, of which were six Caucasian control males aged 42 ± 9.5 years and seven Caucasian females aged 42 ± 9.2 years.

**Table 1:** Clinical and biochemical findings.

**Patients**	**Age**	**Sex**	**Peak**	**PP total (mg/gHb)**	**Proto%**	**Zinco%**	**FECH analysis**
Pt1	36	M	632	123.7	97	3	FECH: c. [1-251G>C; 194+4350_463+1197del5577]; [315-48T>C]
Pt2	32	F	634	63.4	91	9	FECH: c. [215dupT]; [315-48T>C]
Pt3	40	F	634	84.2	39	0	ALAS2: c. [1706_1709delAGTG]; [=]
Pt4	29	M	634	90.4	96	4	FECH: c. [1-251G>C; 194+4350_463+1197del5577]; [315-48T>C]
Pt5	36	M	633	64.4	98	2	FECH: c. [215dupT]; [315-48T>C]
Pt6	45	F	634	63.9	95	5	FECH: c. [901_902delTG]; [315-48T>C]
Pt7	51	F	633	165.4	97	3	FECH: c. [901_902delTG]; [315-48T>C]
Pt8	36	F	635	94.2	95	5	FECH: c. [215dupT]; [315-48T>C]
Pt9	51	M	634	67	96	4	FECH: c. [464–1169 A>C]; [315-48T>C]
Pt10	25	M	632	38	94	6	FECH: c. [215dupT]; [315-48T>C]
Pt11	27	F	631	41	92	8	FECH: c. [215dupT]; [315-48T>C]
Pt12	54	F	634	55	96	4	FECH: c. [464–1169 A>C]; [315-48T>C]
Pt13	24	M	634	63.7	96	4	FECH: c. [67+5G>A]; [315-48T>C]
Pt14	40	M	634	95.7	98	2	FECH: c. [215dupT]; [315-48T>C]
Pt15	27	F	631	61.8	94	6	FECH: c. [1080_1081delTG]; [315-48T>C]
Pt16	49	F	634	29.6	93	7	FECH: c. [215dupT]; [315-48T>C]
Pt17	48	M	631	33.2	92	8	FECH: c. [464–1169 A>C]; [315-48T>C]
Pt18	54	M	635	152.4	98	2	FECH: c. [215dupT]; [315-48T>C]
Pt19	37	F	634	55.7	96	4	FECH: c. [1-251G>C; 194+4350_463+1197del5577]; [315-48T>C]

The EPP exhibited typical plasma fluorescence peak at 631–635 nm. Total erythrocyte PP-IX (with the percentage of PP/ZPP) was increased in all patients, and no differences were observed between males and females (respectively, μ = 77 ± 38 and μ = 76 ± 37). FECH mutation was described for every patient and are reported in Table 1. The controls were tested for plasma peak and the results were negative.

### Increase of Properdin and C5 Levels and Decrease of Inhibitor FH in PP Samples Between Seasons

**Figure 1:**
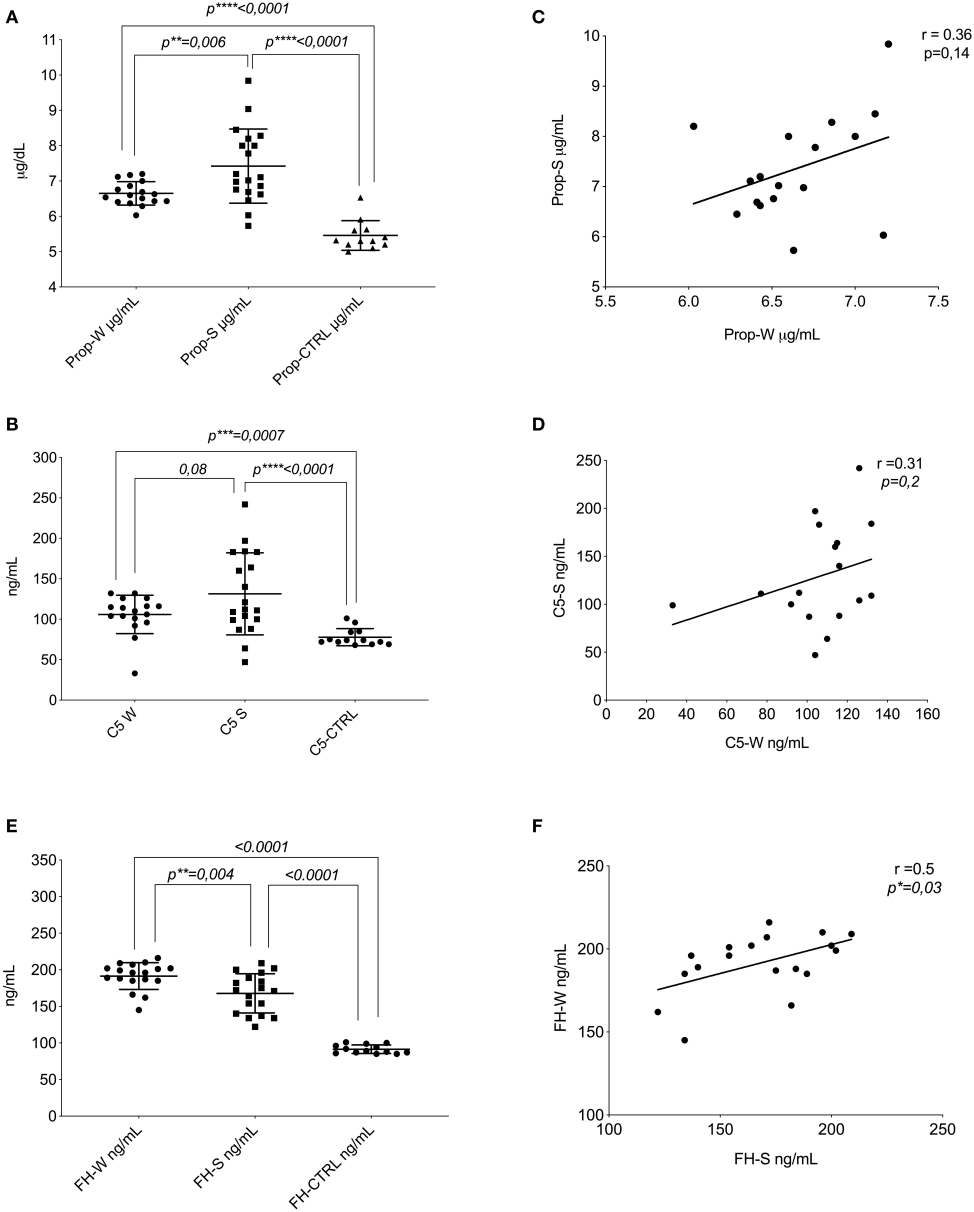
Protein of AP assays. **(A)** Distribution of the mean levels and ±DS of properdin in winter (Prop-W; μ = 6.6 ± 0.33), properdin in summer (Prop-S; μ = 7.4 ± 1.05), and the control group (Prop-CTRL; μ = 5.4 ± 0.42). **(B)** Distribution of the mean levels and ±DS of C5 in winter (C5-W μ = 105.9 ± 23.7), C5 in summer (C5-S μ = 131–3 ± 50), and the control group (C5-CTRL μ = 77.5 ± 10.6). **(C)** Correlation between the seasonal values in patients with PP of properdin (*r* = 0.36, *p* = 0.14). **(D)** Correlation between the seasonal values in patients with PP of C5 (*r* = 0.31, *p* = 0.2). Factor H assay. **(E)** Distribution of the mean levels and ±DS of FH in winter (FH-W μ = 191.4 ± 18.31); FH in summer (FH-S μ = 167.7 ± 26); and the control group (FH-CTRL μ = 91.4 ± 15.9). **(F)** Correlation between the seasonal values in patients with PP for FH (*r* = 0.5, ^*^p = 0.03). FH-S, Factor H-Summer; Prop-W, Properdin Levels in Winter; Prop-S; Properdin Levels in Summer. ^**^P ≤ 0.01; ^***^P ≤ 0.001; ^****^P ≤ 0.0001.

Compared to healthy subjects (Prop-CTRL), marked difference (<0.0001) in properdin levels of patients with PP both in winter (Prop-W) and in summer (Prop-S) were noted. Interestingly, the comparison between Prop-W and Prop-S also revealed a significant increase in properdin during summer (<0.006) (Figure 1A). A statistically significant difference was also observed between C5 levels in winter (C5-W) and summer (C5-S) in patients with PP compared to the controls (*p* < 0.007 and *p* = 0.0001, respectively). A slight increase in C5 was also evident when comparing summer (C5-S) and winter (C5-W) values in patients with PP (*p* < 0.08) (Figure 1B). We also observed a positive correlation tendency between properdin and C5 in both summer and winter (respectively, *r* = 0.36, *p* = 0.14; *r* = 0.31, *p* = 0.2) (Figures 1C,D).

The mean value of FH in winter (FH-W) was higher compared to that in summer (FH-S) in PP subjects (*p* = 0.004). Moreover, the values of patients with PP either from summer or winter were significantly higher compared to healthy controls (FH-CTRL) (<0.0001) (Figure 1E).

The escalation of FH between seasons (winter vs. summer) was associated with a positive intra-patient correlation (*r* = 0.5, *p* = 0.03) (Figure 1F). The Supplementary Data reports the statistical analyses of each protein of AP, stratifying the patient population by sex and age (Supplementary Figures 1, 2). No changes were found in either group.

### Correlation Between the Major Important Proteins of the AP

**Figure 2:**
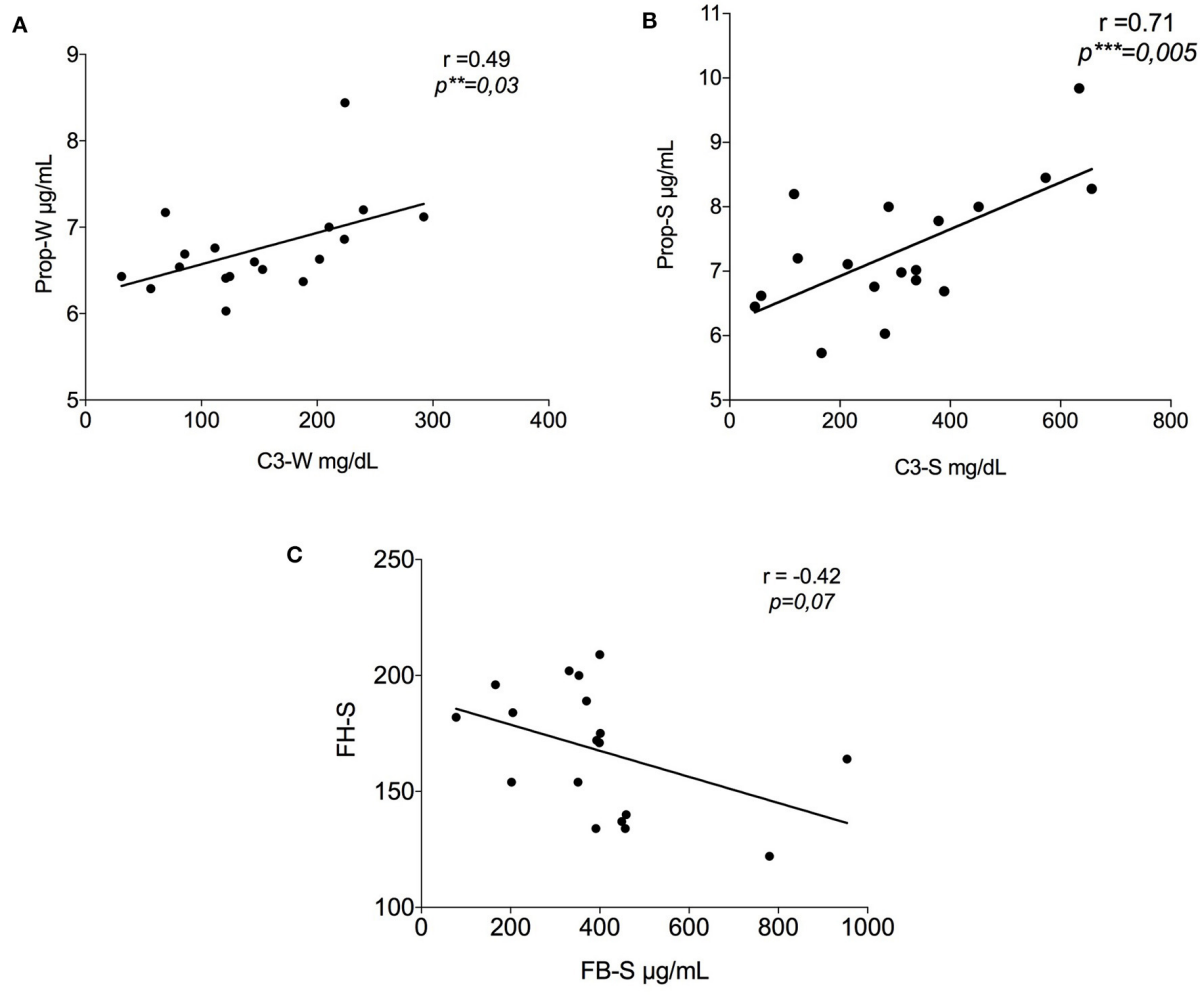
Correlation between the results of the alternative pathway proteins in summer. **(A)** Positive correlation between properdin and C3 in winter (*r* = 0.49, ^**^p = 0.03); **(B)** Positive correlation between properdin and C3 in summer (*r* = 0.71, ^***^p = 0.005); **(C)** Negative tendency between FH-S and FB-S data previously show by Granata et al. (24) (*r* = −0.42, *p* = 0.07). FH-S, Factor H-Summer; FB-S, Factor B-Summer.

Following the same methodology, data about C3 and FB were previously obtained from the same samples (24). Here, we report an additional analysis with data obtained in this study. A significant positive correlation (*r* = 0.49, *p* = 0.03) between C3 and properdin values in winter PP samples was established (Figure 2A). Figure 2B represents the correlation between the same factors in summer, which showed an even stronger positive correlation (*r* = 0.71, *p* = 0.005). On the contrary, a negative correlation was seen between FB-S and FH, i.e., FH increased with a gradual reduction in FB (*r* = −0.42, *p* = 0.07) (Figure 2C).

### Personal Exposure to Global Radiation and Its Relationship With the Complement Metabolites

**Figure 3:**
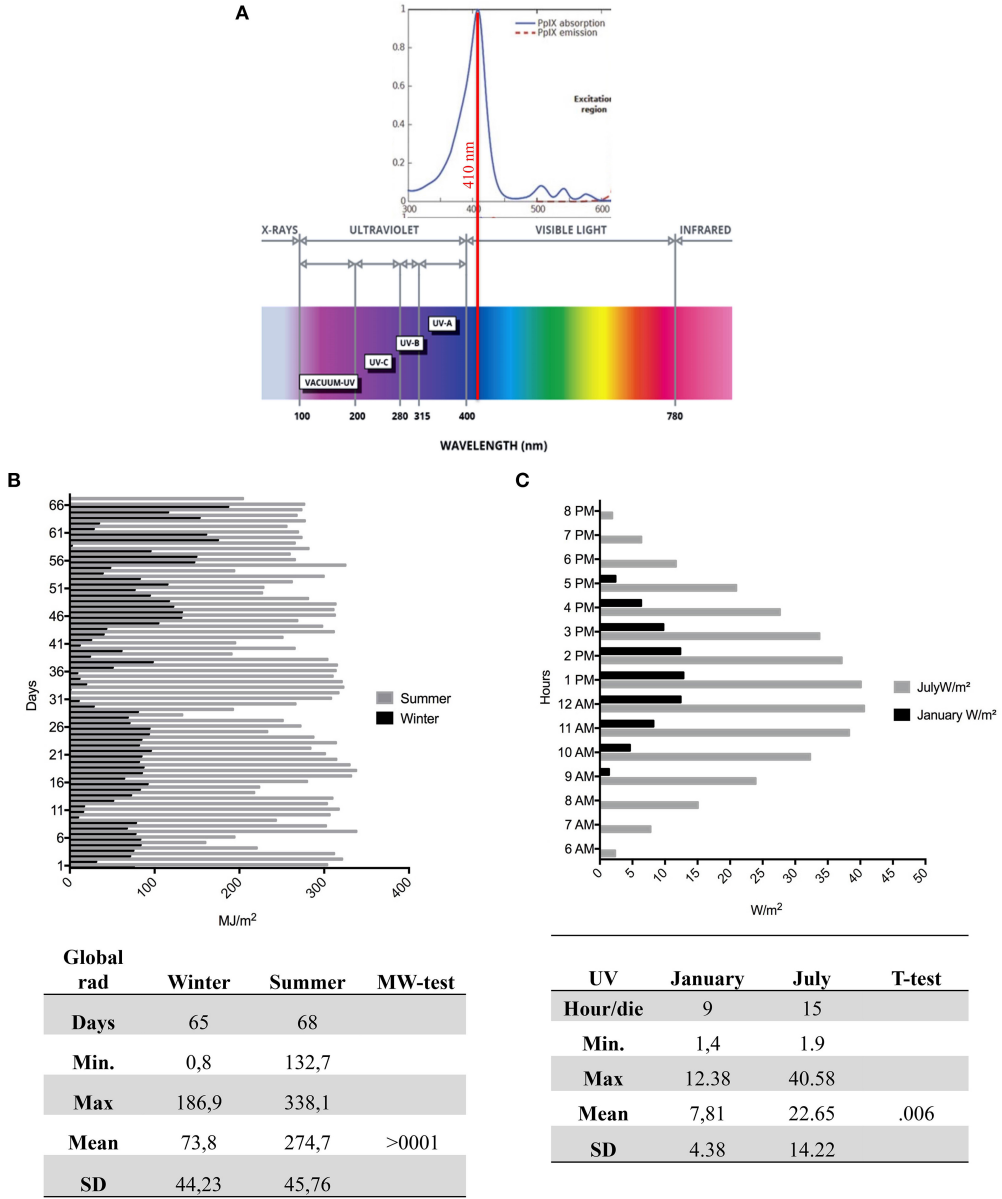
The Global Radiation, UV radiation, and PP-IX peak. **(A)** Upper: The portion of the PP-IX absorption spectrum with a maximum peak around 410 nm in visible. Lower the global radiation spectrum with different wavelengths of UVC (200–280 nm), UVB (280–315 nm), UVA (315 to ≈400 nm), and visible light (≈400–780). **(B)** The daily mean detection by ARPA of global radiation in 65 days in winter (black) and 68 days of summer (gray). The figure shows a high summer trend of global radiation compared to winter. In the figure are sums of the statistical values with a significant mean difference (>0001) between seasons. **(C)** The daily hours in January and in July, during the maximal variability of UV ray. In the figure are sums of the statistical values with a significant mean difference (>0.006) between seasons. PP-IX, Protoporphyrin-IX; ARPA, Regional Agency of Environmental Protection.

Figure 3A represents the radiation spectrum with the PP-IX absorption lambda to 410 nm in visible light. Figure 3B shows different trends of global radiation between the seasons; in winter it was registered for 65 days with a mean and SD of μ = 73.8 ± 44.23, that was statistically different compared to 68 days of summer detection (μ = 247.7 ± 45.76) (*p* < 0.001). Moreover, Figure 3C represents the mean and SD of UVA by hour per day in January (μ = 7.81 ± 4.83) and July (μ = 22.65 ± 22.41).

**Figure 4:**
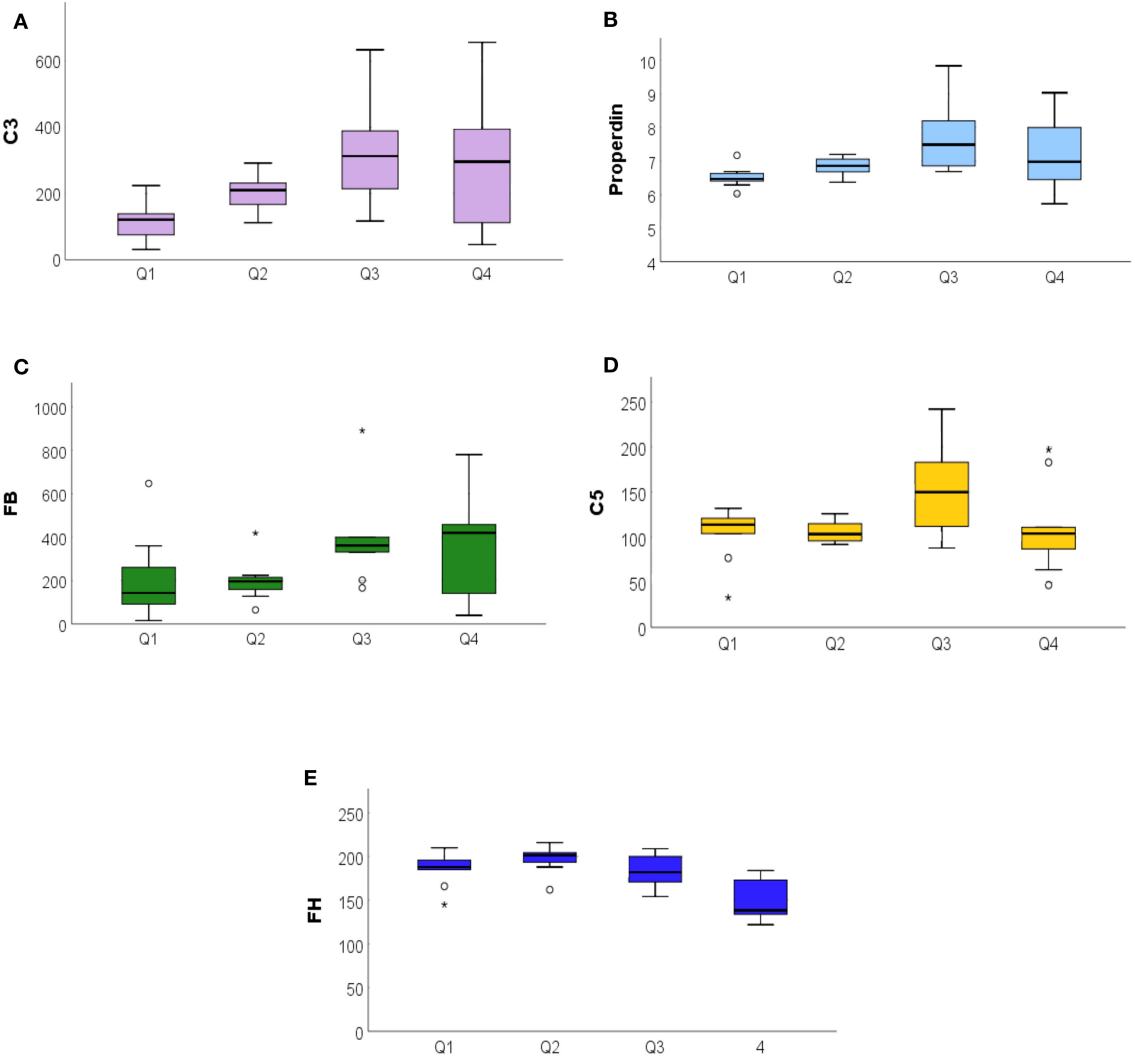
The ANOVA analysis among the exposure to global radiation in the 14 days before blood sampling related to complement system proteins. **(A)** Significative increase of C3 among the quartile (<007), in particular to Q1 from Q3, with a stabilization in Q4. **(B)** Properdin significant increase (<0.016). **(C)** Increased FB values among quartiles (<0.08). **(D)** Stable distribution of C5 (<057). **(E)** FH decreasing among quartiles (<0.001). FB, Factor B. ^*^P ≤ 0.05.

The global radiation collected from ARPA during the 14 days preceding the blood draw was divided into quartiles: Q1 (51.3–65.7 MJ/m^2^), Q2 (75.4–148.6 MJ/m^2^), Q3 (159.5–283.1 MJ/m^2^), and Q4 (286.0–297.9 MJ/m^2^). The distribution of metabolites of the CS in different quartiles of global radiation exposure was depicted using box plots. Comparing the groups, we observe significant or marginally significant differences for the AP metabolites: C3 (*p* = 0.007), properdin (*p* = 0.016), FB (*p* = 0.084), C5 (*p* = 0.057), and FH (*p* < 0.001). C3 and properdin showed a positive increase on exposure to global radiation, while FH showed a decreasing trend. The C5 and FB show marginally significant differences (Figures 4A–E).

## Discussion

The phototoxic reaction is one of the predominant clinical symptoms in PP. The present study highlights that the reaction is not only attributable to a local response in the dermis of the patients but also include a systematic involvement. The symptoms experienced by some patients and described as chills, malaise, fatigue, nausea, excessive temperature sensitivity, or generally unwell after long solar exposure (26, 27) could be attributed to the implication of the immune system.

A combination of multifactorial conditions, including seasons, cloud cover, intensity and the extent of sun exposure, and the time of the day (28), is responsible for the heterogeneity of PP symptoms, whose intensity varies from mild to severe. Furthermore, the patients are aware of the limit beyond which even mild symptoms can occur (29). Therefore, the study of a systemic involvement cannot take place with intensive exposure to sunlight. We used a method that allowed us to analyze the CS fluctuations associated with the variation of light intensity between seasons without exposing the patients to dangerous and painful treatments. In our previous work, we reported a significant increase in C3 and FB proteins, primarily during the summer season, which highlighted the involvement of the AP of the CS in the phototoxic reaction of patients with PP (24).

**Figure 5:**
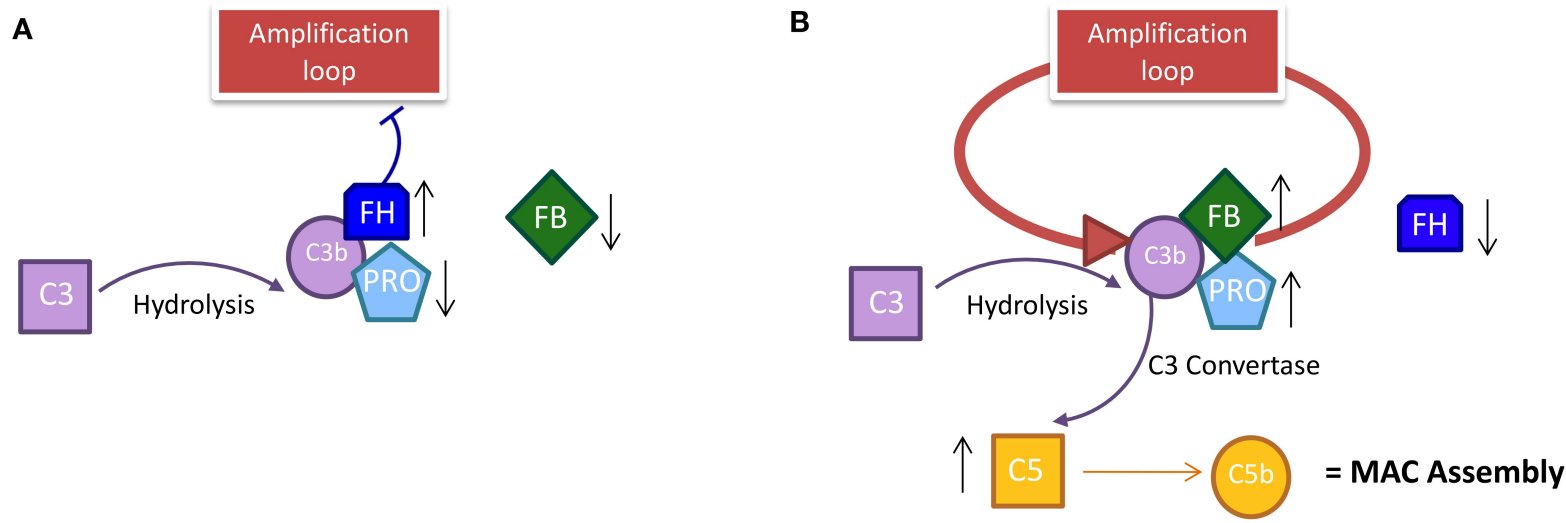
The course of the alternative pathway in PP patients during the season. **(A)** In winter, the decrease in solar irradiation does not trigger the establishment of an amplification loop, because the level of properdin and FB is low. The inhibitor factor H in winter must be high to occupy the C3b site for the spontaneous hydrolysis of C3; this prevents the stabilization of C3 convertase and activates the loop amplification mechanism. **(B)** In summer, the data show the establishment of an amplification loop; the spontaneous hydrolysis of C3 in C3b is stabilized by the increase in properdin and FB that stabilize the C3b in the C3 convertase complex. In summer, the level of inhibitor FH is decreased compared to winter because the link with C3b is already occupied by FB by which it competes. C3b activation leads to the activation of C5 with consequent MAC assembly activation. PP, Protoporphyria; MAC, Membrane Attack Complex; FH, Factor H; FB, Factor B.

Here, we reported additional experiments that strengthen this presumption. Properdin is the main protein that binds the FB-C3b complex, and it triggers the amplification loop in the AP (Figure 5B). Properdin was found to be positively correlated with C3 in summer PP samples (Figure 2B), suggesting the AP stimulation during this season.

However, the PP sample in winter (compared to control) showed enhanced levels of properdin too, suggesting the stimulation of AP also during this season. The exposure of patients to global radiation also during winter (Figure 1B) could justify the above findings. Moreover, few patients may also be sensitive to some types of artificial light that triggers the reaction (30).

Furthermore, with the increase of FB proportional to the decrease of FH, it further supports the theory of the strong and looping solution of AP to light exposure in summer.

The lower levels of FH in summer compared to winter should cause a loss of control over the loop of AP, corroborating the thesis of the AP stimulation. FB competes with FH for the same C3b binding site and its increase is proportional to the FH decrease in PP samples. Taken altogether, this supports the theory of a strong and looping solution of AP to light exposure in summer. The higher level of C5 proteins in summer vs. winter, resulting in the formation of the membrane attack complex (MAC) assembly for cell lysis in summer, also substantiated this evidence.

A differential function of the CS has been already reported in other chronic and acute pathologic conditions (3–5). Thus, similarly, in PP, the excitation of PP-IX should activate both functions of the CS of the AP compared to healthy controls. In winter, the intensity and duration of global radiation (Figures 3B,C) and the less sun-exposed skin areas should lightly stimulate PP-IX, causing only a mild stimulation of the CS (upregulation of FB; Properdin; C5; FH). This should represent the chronic branch of system, which can, in turn, favor the tissue homeostasis and maintain the patient in a free symptoms status. Therefore, AP can respond to the signal for tissue repair and recovery with minimal cell damage in winter (chronic phase) and accordingly acts as the mechanism of alert (12).

On the contrary, in summer, a stronger intensity and longer sun radiation with bigger sun-exposed skin areas could acutely activate the AP. The enhanced light intensity and exposure in summer causes a rapid increase of AP proteins and a decrease of the inhibitor FH. The loss of inhibition activates the AP loop and an acute response, resulting in tissue destruction and aggravation of symptoms that can last up to 10 days (31). The decay of excited PP-IX is quite fast; the visible light absorption creates a singlet-excited state in PP-IX (1PP^*^), with nanosecond lifetime, which undergoes a fast relaxation to excited triplet state (3PP^*^) with duration of milliseconds. During this time, the PP-IX molecule transfers its energy to molecular oxygen (O_2_) with a consequent decay of excited PP-IX. Therefore, the long lifetime of symptoms could be better justified by the implication of systemic answer and could not be due to the time of decay of excited PPIX (32). It is also well-known that the levels of complement proteins may depend on sex and age (33). We excluded this variability in our population, stratifying it for sex and age and analyzed for each protein of AP (Supplementary Data).

Moreover, all assumptions are supported by the evaluation of exposure to global solar radiation. As the global radiation increased, the main factors of the AP, such as C3 and properdin, increased as well. Since the FH values which were divided into quartiles decreased with a concomitant increase of global radiation in Q3 and Q4, this confirms the loss of inhibition of the AP loop in summer. The protein C5 and FB showed marginally significant differences, but with an increasing tendency throughout the quartiles. This uneven growth could be explained by the fact that the method is not designed on patients but represents a collection of global radiation values during the 14 days prior to the blood draw. During this period, some patients may not have been fully exposed. It is well-known that patients with PP change their behavior during the summer season to avoid sun exposure and the maximum of the symptoms, minimizing the possible acute stages of the disease (34).

To improve the quality of life of the patients for several diseases, the management of symptoms and pain is of utmost importance (35, 36). Likewise, for PP, a constant challenge prevails in daily life to avoid the pain that leads to reduced quality of life (37). Moreover, analgesic drugs fail to relieve the pain in PP (31).

In conclusion, the new insights provided by this study about the involvement of the systemic response in PP can help to understand anti-inflammatory mechanisms of existing drugs, such as alpha-melanocyte–stimulating hormone (α-MSH), or to identify new therapeutic targets that are able to interrupt the systemic response in EPP or that are useful for pain management in photodynamic therapy (PDT) (32, 38).

Future study will be necessary for personalizing the detection method of visible light exposure. At the same time, a more suitable *in vitro* setting to evaluate the level of AP activation during different timelines of light exposure should be developed, thus avoiding the involvement of the patient in an invasive treatment. This will provide a deeper insight of the clinical symptoms complained by patients, such as malaise, after a long sun exposure.

## Data Availability Statement

The raw data supporting the conclusions of this article will be made available by the authors, without undue reservation.

## Ethics Statement

The studies involving human participants were reviewed and approved by according to the World Medical Association Declaration of Helsinki for medical research, all subjects involved in this study signed informed consent for the diagnosis and research approved by the ethics committee of our institution, Fondazione IRCCS Ca' Granda–Ospedale Maggiore Policlinico and the identity of the study participants were anonymized. The patients/participants provided their written informed consent to participate in this study.

## Author Contributions

FG designed the study, performed statistical analysis, and wrote the manuscript. LD performed the ELISA experiments. VB carried out a genetic diagnosis of patients. SF managed the toxicology lab of Policlinico and suggested the method for global radiation and UV detection from ARPA data. GD and GG recruited the patients. IM oversaw the work. ED critically revised the results and manuscript. The manuscript has been read and approved for submission by all authors.

## Conflict of Interest

The authors declare that the research was conducted in the absence of any commercial or financial relationships that could be construed as a potential conflict of interest. The reviewer AM declared a shared affiliation with the authors SF and IM to the handling editor at the time of review.
