# Supplementary material for: Alternative Pathway Involvement in Protoporphyria Patients Related to Sun Exposure
Source: Front Immunol. 2021 Feb 16;12:615620. doi: 10.3389/fimmu.2021.615620 (PMC7921788; doi:10.3389/fimmu.2021.615620)
Supplement: Supplementary file 1 [file Data_Sheet_1.PDF]

Supplementary data

It is well known that the levels of complement proteins may depend on age and sex. In order to exclude this variability, the data of EPP patients were divided and analysed for each protein of AP.
Supplemental figure 1 shown the differentiation for sex and supplemental figure 2 the classification based on two aged groups: class-I 23-36 years in bold and class-II 39-50 years (Figure 2).
The results showed that sex and age did not interfere with the analysis, as described in Figure 1. The differences between males and females decrease more in summer reinforced our hypothesis that in these patients it is the excitation of the PPIX that produced an increase of the complement during the season. While, as regarding age figure 2 shown an equal heterogeneity into age classes.

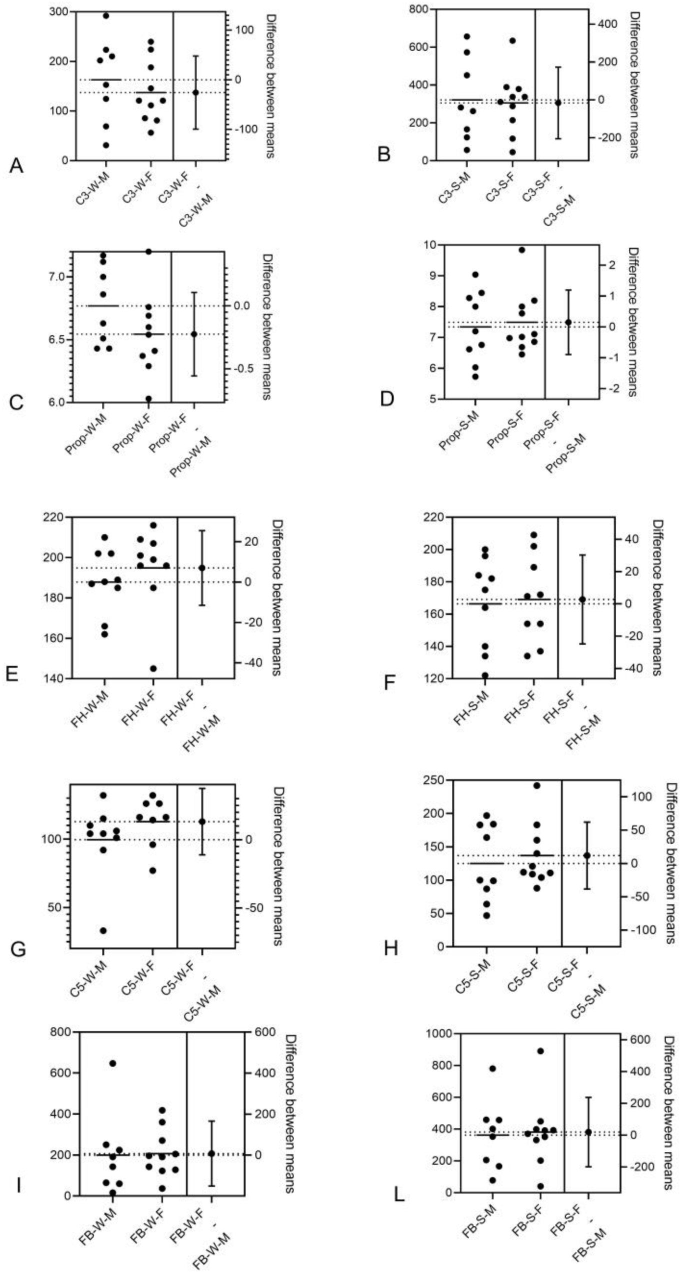

Supplemental Figure 1: **AP assays in male and female during season**. ***a.*** Estimation plot of distribution of C3 in winter for male (μ = 163.2 ± 81.11) and female (μ = 137.5 ±61.71) without difference between groups (p = 0.47). ***b.*** Estimation plot of distribution of C3 in summer for male (μ = 321.4 ± 217.4) and female (μ = 305.4 ± 161.3) without difference between groups (p = 0.85). ***c.*** Estimation plot of distribution of properdin in winter for male (μ = 6.7± 0.3) and female (μ = 6.5 ±0.33) without difference between groups (p = 0.17). ***d.*** Estimation plot of distribution of properdin in summer for male (μ = 7.3± 1.5) and female (μ = 7.5 ±1) without difference between groups (p = 0.77). ***e.*** Estimation plot of distribution of FH in winter for male (μ = 187.9 ± 16) and female (μ = 194.9 ±20.7) without difference between groups (p = 0.43). ***f***. Estimation plot of distribution of FH in summer for male (μ = 166.3± 28.1) and female (μ = 6169.1 ±27) without difference between groups (p = 0.83).
g. Estimation plot of distribution of C5 in winter for male (μ = 99.7 ± 27.3) and female (μ = 112.9 ±18.14) without difference between groups (p = 0.26). ***h.*** Estimation plot of distribution of C5 in summer for male (μ = 125 ± 57.1) and female (μ = 137 ± 46.6) without difference between groups (p = 0.62). ***i.*** Estimation plot of distribution of FB in winter for male (μ = 199.5± 199) and female (μ = 207 ±114) without difference between groups (p = 0.91). ***l.*** Estimation plot of distribution of FB in summer for male (μ = 362.4± 219) and female (μ = 215 ±68) without difference between groups (p = 0.85).

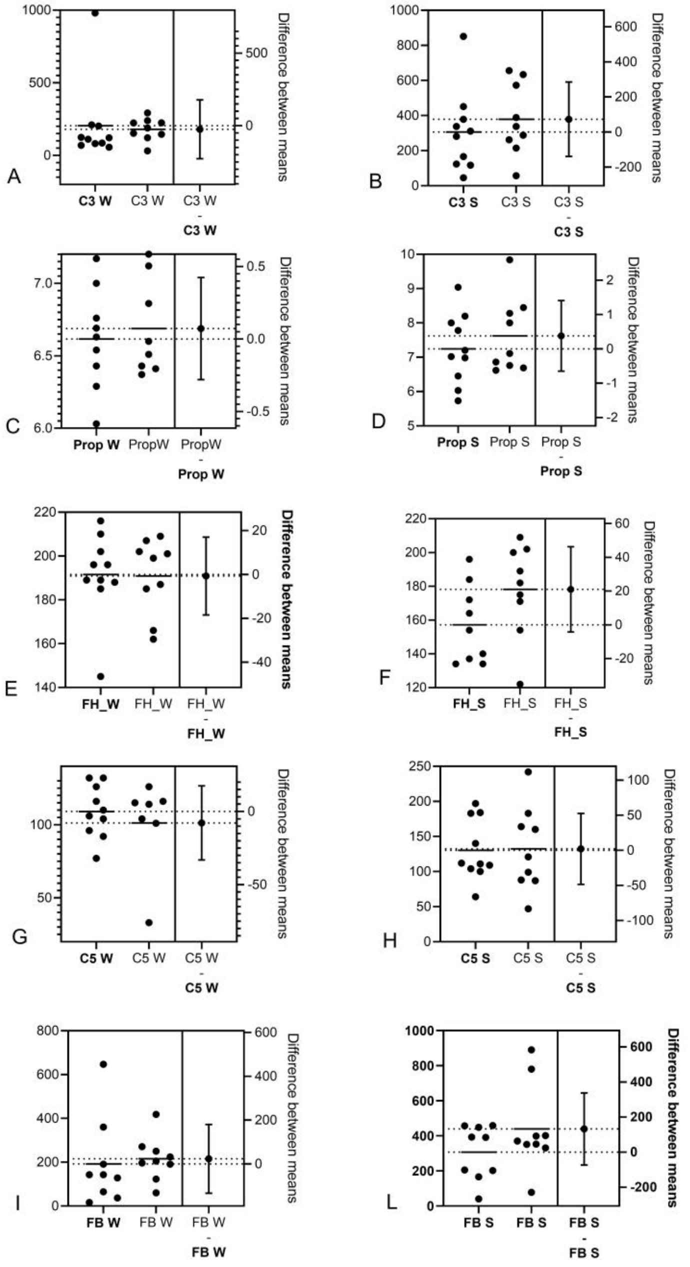

Figure 2**AP assays in two age classes: I (23-36 years in bold) and II (39-50 years)** ***a.*** Estimation plot of distribution of C3 in winter in class-I (μ = 118 ± 55.1) and class-II (μ = 179 ± 78.2) without difference between groups (p = 0,1). ***b.*** Estimation plot of distribution of C3 in summer for class-I (μ = 246 ± 138) and class-II (μ = 378 ± 204) without difference between groups (p = 0.12). ***c.*** Estimation plot of distribution of properdin in winter for class-I (μ = 6.6± 0.34) and class-II (μ = 6.7 ±0.33) without difference between groups (p = 0.66). ***d.*** Estimation plot of distribution of properdin in summer for class-I (μ = 7.3± 1) and class-II (μ = 7.6 ±1.1) without difference between groups (p = 0.44). ***e.*** Estimation plot of distribution of FH in winter for class-I (μ = 191.6 ± 19.2) and class II (μ = 191 ±17.3) without difference between groups (p = 0.93). ***f.*** Estimation plot of distribution of FH in summer in class-I (μ = 166.3± 28.1) and class-II (μ =178 ±27.2) without difference between groups (p = 0.1). ***g.*** Estimation plot of distribution of C5 in winter for class-I (μ = 109 ± 18) and class-II (μ = 101.3 ±31) without difference between groups (p = 0.9). ***h.*** Estimation plot of distribution of C5 in summer for class-I (μ = 130.4 ±44) and female (μ = 132 ± 60.1) without difference between groups (p = 0.93). ***i.*** Estimation plot of distribution of FB in winter for class-I (μ = 192.2± 198) and female (μ = 215 ±99.4) without difference between groups (p = 0.75). ***l.*** Estimation plot of distribution of FB in summer for class I (μ = 306.9± 155) and class-II (μ = 430 ±240) without difference between groups (p = 0.2).

**Controls**	**Age**	**Sex**	**Plasma Peak**
**C1**	36	M	Negative
**C2**	30	M	Negative
**C3**	47	F	Negative
**C4**	54	F	Negative
**C5**	52	M	Negative
**C6**	39	F	Negative
**C7**	38	M	Negative
**C8**	47	M	Negative
**C9**	46	F	Negative
**C10**	46	M	Negative
**C11**	43	F	Negative
**C12**	19	F	Negative
**C13**	51	F	Negative

Table 1: Clinical and biochemical findings of control samples. Six Caucasian CTRL male aged 42±9.5 years and seven Caucasian females aged 42±9.2 years.
